# Drawing It Out: A Descriptive Study on How Medical Students Use Graphic Medicine to Depict Their Transition to Third Year

**DOI:** 10.1007/s40670-025-02377-w

**Published:** 2025-04-07

**Authors:** Casey Fishman, Justin Do, Fred Markham

**Affiliations:** Sidney Kimmel Medical College at Thomas Jefferson University, 1000 Walnut Street, Philadelphia, PA 19107 USA

**Keywords:** Graphic medicine, Empathy, Imposter syndrome, Clinical rotations

## Abstract

**Purpose:**

Graphic medicine provides a unique method for medical students transitioning to clinical rotations to express their sentiments.

**Materials and Methods:**

In total, 36 cartoons created by 248 third-year medical students to represent their transition to clinical rotations in 2018–2019 were qualitatively analyzed for tone and common thematic elements.

**Results:**

Twenty-four (67%) of these cartoons were coded as negative, eight (22%) were mixed, and four (11%) were positive. Common themes present were the use of horror imagery, feelings of inadequacy, and time pressure.

**Discussion:**

Analysis of graphic medicine provides educators an opportunity to reflect and incorporate ways to address the problems highlighted.

## Introduction

Clinical rotations are a key aspect of undergraduate medical education. However, the transition between pre-clinical and clinical curriculum often presents challenges. Medical students often feel unprepared for clinical rotations, with a study finding that only 43% of them felt prepared with core clinical skills [1]. Given these challenges, it is important to investigate medical students’ perceptions of their clinical education to identify unnecessary stressors on students.

The field of graphic medicine shows great promise in reflecting individual’s beliefs and experiences through visual mediums both as an education tool and as a mode of communication [2], presenting an effective method of gathering unstructured qualitative information. It presents a novel way for medical students to express their concerns to faculty outside of traditional surveys and written text. Previous works have shown the value of comics in building professional and personal resilience in medical students [3] and familiarizing students with risk-taking and failure in a safe environment [4].

The goal of this study was to investigate medical student attitudes during the transition between the pre-clinical and clinical sections of their undergraduate curriculum using graphic medicine.

## Methods and Materials

Reflection rounds were introduced at an urban medical school during the family medicine rotation from 2018 to 2019 [5]. These rounds consisted of four 1-h sessions where 248 third-year medical students reflected on their emotional state and the transition to clinical years. During the final session, the students created 39 cartoons depicting a variety of themes relating to their sentiments towards the journey of medical school at that junction.

The cartoons were qualitatively analyzed using both images and text included in the cartoon. The cartoons were coded based on whether they expressed an overall positive, negative, or mixed outlook. Out of the 39 cartoons, three could not be coded as they did not have an emotional component or could not be understood.

In addition to coding them according to the general emotional outlook, the cartoons were further divided into subdomains of themes, including infantilization, time, stages of medical education, intimidation from attendings, negative hierarchy, incompetence, and comparison to non-medical careers.

## Results

**Fig. 1:**
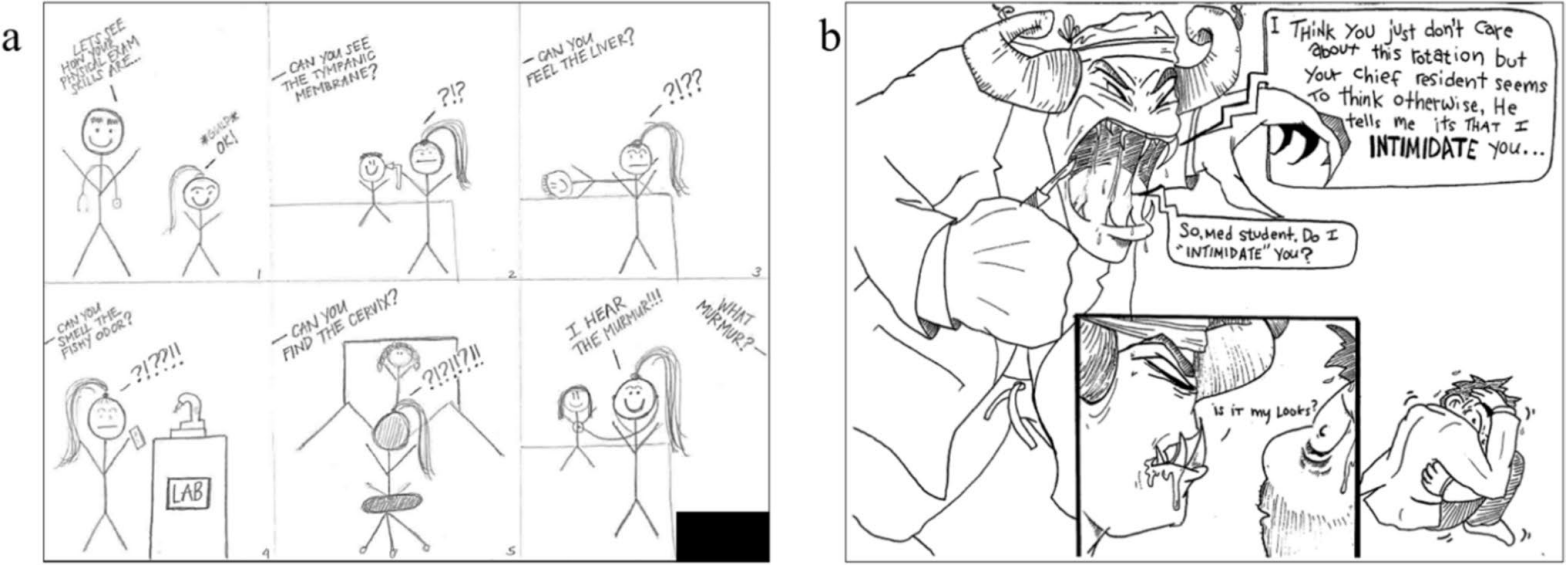
Comics by medical students about incompetence and power gradient

Of the 36 cartoons included in the analysis, four (11%) were positive, eight (22%) were mixed, and 24 (67%) depicted negative emotions. Of the negative cartoons, the most common subdomains present were incompetence, negative hierarchy and culture, and a lack of time. The most prevalent subdomain in the negative cartoons was incompetence, specifically students’ negative impressions of their own capabilities. One cartoon depicts a student practicing auscultation (Fig. 1a), failing to hear abnormal heart sounds multiple times. Ironically, the moment they claim to hear an abnormal heart sound, the murmur is, in fact, absent, suggesting a sense of incompetence and futility among the medical students.

Several of the negative cartoons presented the negative effects of the medical hierarchy by depicting seniors using horror imagery. One cartoon depicts an attending, presumably a surgeon given the scrub cap, mask, and scalpel, as a monster with horns and teeth towering over a cowering medical student rhetorically asking if the student is intimidated (Fig. 1b). The cartoon may have fantastical imagery, but the attending inspires such fear in the student that they had chosen to portray them inhumanely.

**Fig. 2:**
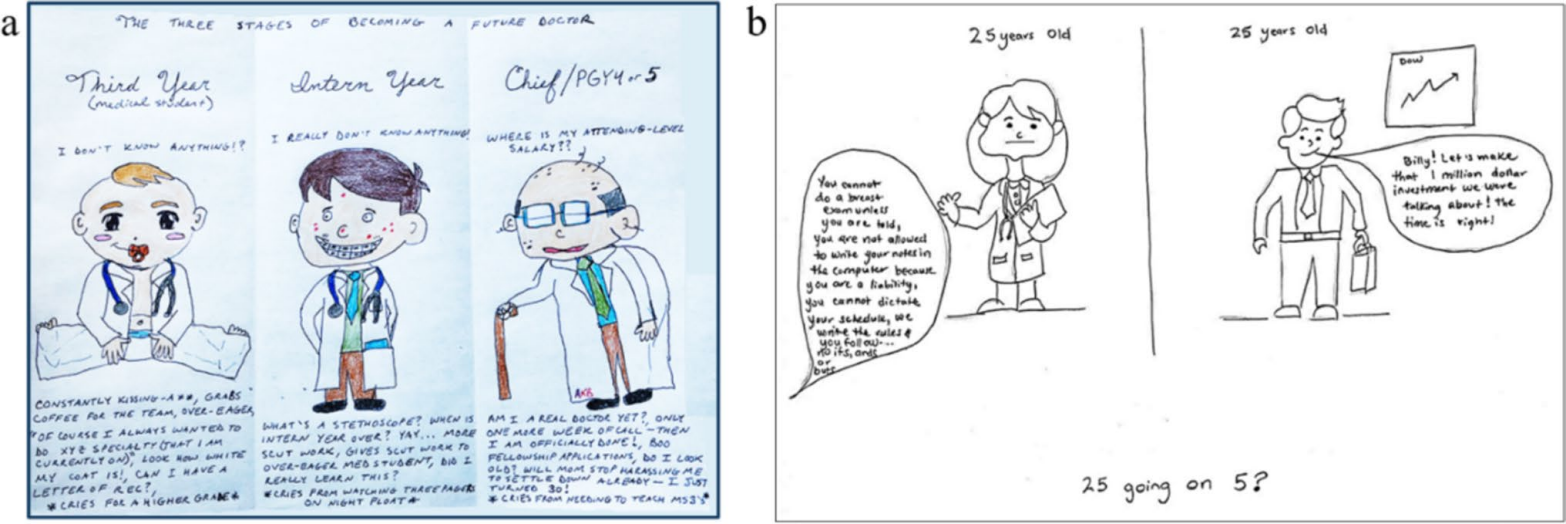
Comics by medical students about infantilization of medicine

The hierarchy of medicine is sometimes critiqued in more direct ways. Another cartoon presents the three stages of becoming a doctor, representing each stage quite unappealingly, first as a baby, then as an enthusiastic child, and finally as a decrepit older adult (Fig. 2a). The captions underscore what the student views as a vicious cycle, where the baby (a medical student) cries for a higher grade, the intern gives the medical student scut work, and the final year resident is just waiting for the promised “attending year salary.”

Sometimes, visual illustrations can embody multiple themes; for instance, a cartoon compares medical education to non-medical careers, underscoring the infantilization present in medical education (Fig. 2b). The medical student is told that they are not trusted to do anything, while their peer in a business field has just had their plan approved by a senior. No doubt, the illustrator of this cartoon may be looking at their non-medical peer quite enviously.

**Fig. 3:**
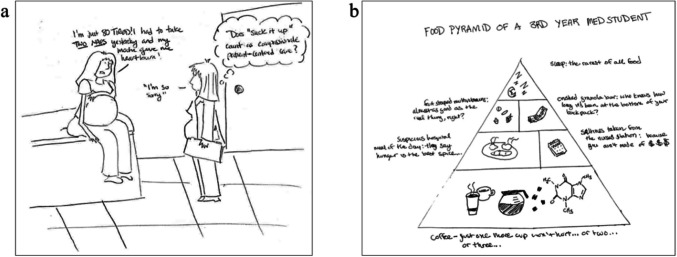
Comics by medical students about a lack of time and self-care

Another common negative theme expressed is lack of time. A cartoon depicts an internally fuming pregnant medical student (Fig. 3a) listening to a pregnant patient complaining about being tired after taking two naps. The medical student seems to be losing her empathy as although she and the patient are in similar situations, the student does not have enough time to rest due to the conditions of the medical training system.

Students not only highlighted the lack of personal time but the unhealthy coping mechanisms that they must undertake. One cartoon (Fig. 3b) demonstrates the food pyramid of a third-year medical student, highlighting coffee as a foundational food group and sleep being “the rarest of all food.”

**Fig. 4:**
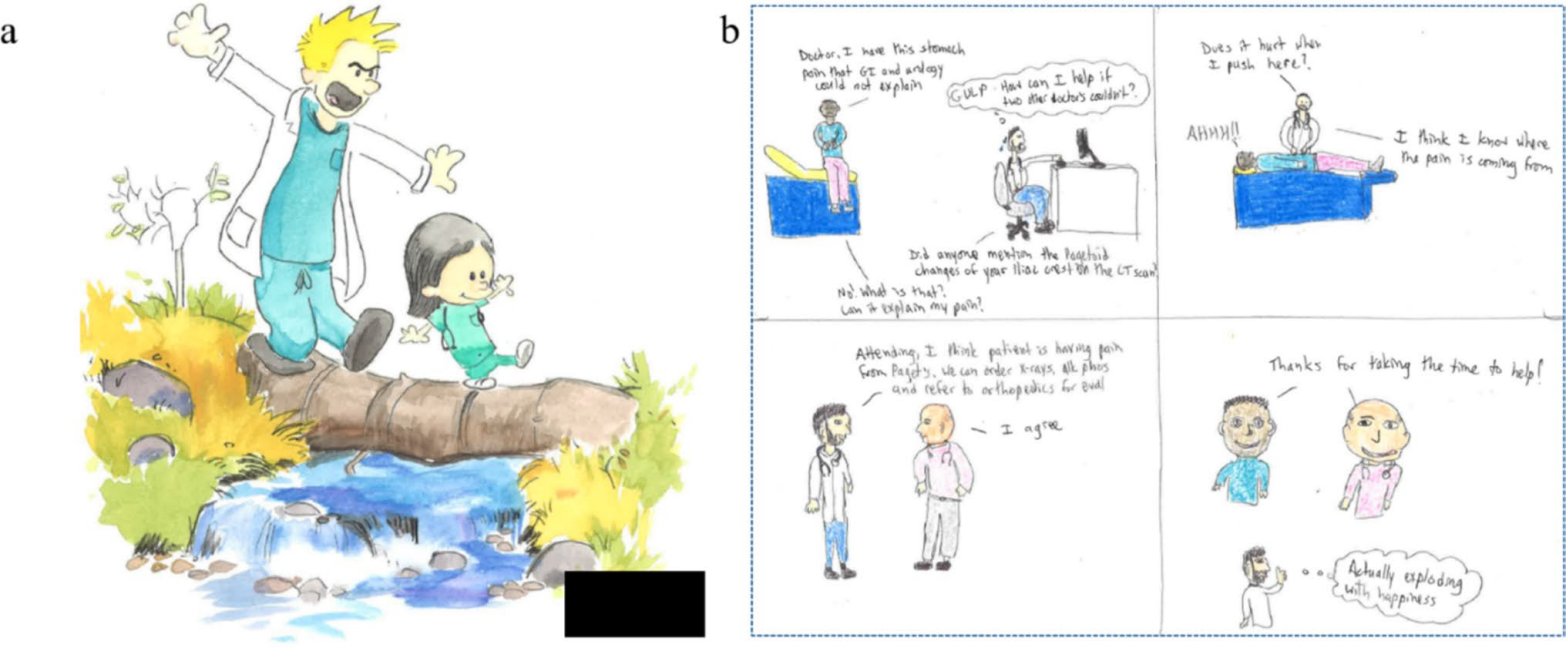
Comics by medical students depicting positive interactions with teachers

Although students used graphic medicine to depict mostly negative emotions, positive comics often focused on the role of supportive mentors and teachers. One of the positive cartoons depicted a relationship between an attending and a medical student that gave them the confidence to learn and grow, basing the imagery on a *Calvin and Hobbes* cartoon (Fig. 4a). This contrasts with many other cartoons that depict the hierarchy of medicine negatively and demonstrates how a good relationship with a supervisor can completely shape a student’s outlook on medical training. Another positive cartoon demonstrated an attending who agreed with the medical student’s judgment and thanked the student for taking the time to help (Fig. 4b). This echoes the importance of positive verbal feedback, which can help facilitate a student’s confidence in making medical decisions.

## Discussion

The utilization of graphic medicine provides a unique insight into current issues within the medical training system and how it affects students. Many themes identified through these comics are supported by previous works using other mediums. A study found a direct correlation between the usage of class ranking and the prevalence of imposter syndrome [6]. As seen in multiple cartoons that embodied the subdomain of depicting superiors as horror figures, a power hierarchy in medicine has negative psychological effects on trainees. A study similarly found that medical students illustrated their mentors as fiendish monsters and the workplace as a dungeon [7]. Students have been shown to resent their low status in the hierarchal nature of medical training [8], and dysfunctional medical hierarchies have been shown to reduce empathy and lower training value [9]. A negative experience with criticism from more experienced faculty may lead to regret in choosing the medical profession (Fig. 2), which was demonstrated by an Australia-based study [10].

A systematic review of the literature found ample evidence that empathy in medical students declines during the transition to clinical years, explained by a hidden curriculum that presents new stressors on students [11]. A 2018 study found that the self-reported greatest stressors of medical students included medical school workload, performance pressure, time constraints, and lack of balance [12]. As demonstrated in the cartoons, there is a dichotomy between the advice the students give patients and how they are treated, which can create cognitive dissonance and lead to difficulty in performing their duties as medical professionals.

Our study revealed positive aspects of clinical rotations for undergraduate mentorship. Positive mentorship was displayed using bright, cheerful colors, demonstrating the professional and personal benefits of a positive learning environment. Successful medical student mentorship programs have improved specialty recruitment and retention, department and student research productivity, and students’ professional development [13]. Positive role models have been shown to positively impact student’s views on medicine, assist in the transition between being a medical student and a resident, and reduce stress [14]. This suggests that successful, functional medical teaching models have role models that guide students during their professional development and opportunities for medical students to practice their skills successfully and feel empowered.

While many of the lessons presented in this study are known, the format of graphic medicine provided a less structured, qualitative way for students to express their lived experiences compared to surveys or text. For example, the use of horror imagery more acutely displays the intimidation students may feel due to the power gradient of medical teams, allowing educators to better understand the student perspective. Graphic medicine may present a non-confrontational avenue of communication between students and faculty.

The issues present in this study can be addressed by direct intervention from students. Tangible actions, such as a reduction in non-essential required events, may promote a healthier work-life balance. The power gradient explored by these student comics can be addressed by emphasizing positive mentorship and clear expectation setting. Most importantly, this study highlighted the insight that graphic medicine can bring to the lived student experience, which educators can apply in their practice to foster a more positive environment.

## Data Availability

The original comics are available from the corresponding author by reasonable request.
